# A minimum data standard for wildlife disease research and surveillance

**DOI:** 10.1038/s41597-025-05332-x

**Published:** 2025-06-21

**Authors:** Collin J. Schwantes, Cecilia A. Sánchez, Tess Stevens, Ryan Zimmerman, Greg Albery, Daniel J. Becker, Cole B. Brookson, Rebekah C. Kading, Carl N. Keiser, Shashank Khandelwal, Stephanie Kramer-Schadt, Raphael Krut-Landau, Clifton McKee, Diego Montecino-Latorre, Zoe O’Donoghue, Sarah H. Olson, Mika O’Shea, Timothée Poisot, Hailey Robertson, Sadie J. Ryan, Stephanie N. Seifert, David Simons, Amanda Vicente-Santos, Chelsea L. Wood, Ellie Graeden, Colin J. Carlson

**Affiliations:** 1 Department of Epidemiology of Microbial Diseases, Yale University, New Haven, CT USA; 2 Center for Global Health Science and Security, Georgetown University, Washington, DC USA; 3 Department of Biology, Georgetown University, Washington, DC USA; 4 School of Biological Sciences, University of Oklahoma, Norman, OK USA; 5 Département de Sciences Biologiques, Université de Montréal, Montreal, QC Canada; 6 Center for Vector-borne Infectious Diseases, Department of Microbiology, Immunology, and Pathology, Colorado State University, Fort Collins, CO USA; 7 Emerging Pathogens Institute, University of Florida, Gainesville, FL USA; 8 Blue Tiger, LLC, Timonium, MD USA; 9 Department of Ecological Dynamics, Leibniz Institute for Zoo and Wildlife Research, Berlin, Germany; 10 Department of Epidemiology, Johns Hopkins Bloomberg School of Public Health, Baltimore, MD USA; 11 Wildlife Conservation Society, Health Program, New York, NY USA; 12 Department of Ecology and Evolutionary Biology, Tulane University, New Orleans, LA USA; 13 Paul G. Allen School for Global Health, University of Washington, Pullman, WA USA; 14 Department of Anthropology, Pennsylvania State University, State College, PA USA; 15 School of Aquatic and Fishery Sciences, University of Washington, Seattle, WA USA; 16 Massive Data Institute, Georgetown University, Washington, DC USA

**Keywords:** Ecological epidemiology, Microbial ecology

## Abstract

Rapid and comprehensive data sharing is vital to the transparency and actionability of wildlife infectious disease research and surveillance. Unfortunately, most best practices for publicly sharing these data are focused on pathogen determination and genetic sequence data. Other facets of wildlife disease data – particularly negative results – are often withheld or, at best, summarized in a descriptive table with limited metadata. Here, we propose a minimum data and metadata reporting standard for wildlife disease studies. Our data standard identifies a set of 40 data fields (9 required) and 24 metadata fields (7 required) sufficient to standardize and document a dataset consisting of records disaggregated to the finest possible spatial, temporal, and taxonomic scale. We illustrate how this standard is applied to an example study, which documented a novel alphacoronavirus found in bats in Belize. Finally, we outline best practices for how data should be formatted for optimal re-use, and how researchers can navigate potential safety concerns around data sharing.

## Introduction

Infectious disease is a widely studied topic in wildlife biology and ecosystem science^1^. Every year, countless scientific studies report new data on the prevalence of macroparasites (e.g., ticks and tapeworms) and microparasites (e.g., bacteria, viruses, and other classically defined “pathogens”), hereafter “parasites” for simplicity^2^, in wild animals. These datasets are incredibly valuable, and – especially in aggregate – can be used to test ecological theory^3^; monitor the impacts of climate change^4,5^, land use change^6,7^, and biodiversity loss^8^; and even track emerging threats to human and ecosystem health^9–11^.

Disease ecologists engaged in synthesis research are often faced with reconciling datasets that vary greatly in their scope and granularity. For example, many studies do not report information about sampling effort over space and time, and may not even report the location of sampling sites^9,12^. Similarly, researchers often collect a wealth of host-level data that might help to understand infection processes (e.g., sex, age, life stage, or body size). However, many studies only provide summary statistics for parasite prevalence across different sites, species, or time points, which cannot be disaggregated back to the host level. For example, out of 110 studies we recently reviewed^9^ that have tested wild bats for coronaviruses, 96 only reported data in a summarized format (see Supplemental File 4). When studies did share individual-level data, they often did so only for positive results (11 of 14 studies), making it impossible to compare prevalence across populations, years, or species.

To address these issues, wildlife disease ecology would benefit from best practices for dataset standardization and sharing, similar to those that have been developed for other types of foundational data in the biological sciences^13–15^. Data standards facilitate the sharing, (re)use, and aggregation of data by humans and machines through the use of a common structure, set of properties, and vocabulary. Here, we designed a simple and flexible minimum data standard that is intended to be accessible to a range of practitioners, while providing sufficient structure for large-scale data analysis and meeting expectations for Findable, Accessible, Interoperable, and Reusable (FAIR) research practices^16^. We describe the required properties and structure for wildlife disease data that conform to the standard, building on a set of similar templates for sharing datasets related to arthropod disease vectors^17–20^ that focus on utility and ease of use. We document the development of the data standard, show how it can be applied to a simple dataset reporting coronavirus detection in wild bats, and suggest additional best practices for data sharing.

## Methods

Our goal in this project was to develop guidelines for how researchers can collect and share standardized, well-documented wildlife disease datasets, with a focus on documenting sampling methods and findings. We developed our data standard based on: (i) experience conducting and publishing wildlife disease research, and collaborating with government programs doing the same; (ii) common practices already followed by most scientists in the literature when sharing disaggregated data, including the decisions made by major data sources such as the USAID PREDICT 2 project’s data release^21^; (iii) best practices for sharing ecological data that minimize room for error or loss of data^22–27^; and (iv) interoperability with standards used by other platforms, such as the Global Biodiversity Information Facility (GBIF)^27^. We assumed that parasite genetic sequence data and associated types (e.g., metatranscriptomes) are already widely archived on platforms like NCBI’s GenBank and Sequence Read Archive (SRA), following a different set of best practices, and are unlikely to be stored in the same data structure as we describe here.

The guiding philosophy of the data standard is that researchers should share their raw wildlife disease data in a format that data scientists refer to as “rectangular data” or “tidy data”^28^, where each row corresponds to a single measurement, here meaning the outcome of a diagnostic test. Tests, samples, and individual animals can each have many-to-many relationships due to common practices such as repeated sampling of the same animal, confirmatory tests, or sequencing of samples that test positive, and pooling of samples (sometimes from multiple animals and locations) for a single test. Based on this, there are three main categories of information collected: sample data, host animal data, and the parasite data itself, including both test results and any data characterizing a parasite once it has been detected (e.g., GenBank accession). We developed the fields associated with each of these categories through an iterative process using real-world data, as part of the ongoing development of a new dedicated platform for wildlife disease data, the Pathogen Harmonized Observatory (PHAROS) database (pharos.viralemergence.org). Project-level metadata was developed using the DataCite Metadata Schema as recommended by the Generalist Repository Ecosystem Initiative^29,30^.

## Results

### When to use the data standard

Before applying this standard, we encourage researchers to verify that their dataset describes wild animal samples that were examined for parasites, accompanied by information on the diagnostic methods used and the date and location of sampling. Examples of project types that would be suitable for the data standard include, but are not limited to: the first report of a parasite in a wildlife species^31^; investigation of a mass wildlife mortality event^32^; longitudinal, multi-site sampling of multiple wildlife species for a parasite^33^; regular parasite screening in a single monitored wildlife population^34^; screening of wildlife during an investigation of a human disease outbreak^35^; or a passive surveillance program that tests wildlife carcasses submitted by the public^36^.

Some closely-related types of data are better documented using a different data standard: for example, records of free-living macroparasites (e.g., tick dragging data) can be stored in Darwin Core format like any other biodiversity dataset^27,37^, or can adhere to the MIReAD (Minimum Information for Reusable Arthropod Abundance Data) data standard, which was designed with disease vector surveillance in mind^19^. Similarly, arthropod blood meal datasets can follow another recently-published data standard^18^. Finally, environmental monitoring datasets (e.g., soil, water, or air microbiome metagenomics) not associated with a specific animal under direct or indirect observation should also be handled following other best practices^38,39^.

### The data standard

Our proposed data standard includes 40 core fields (11 related to sampling, 13 related to the host organism being sampled, and 16 related to the parasite itself) and 24 fields related to project metadata. The contents of the 40 core fields and their interpretation are described in Tables 1–3 (split into three tables for the reader’s ease).

**Table 1:** Data standard field definitions (part 1): sampling information.

Variable	Type	Required	Descriptor
Sample ID	String	** ✓**	A researcher-generated unique ID for the sample: usually a unique string of both characters and integers (e.g., “OS BZ19-114” to indicate an oral swab taken from animal BZ19-114; see worked example below), to avoid conflicts that can arise when datasets are merged with number-only notation for samples. Ideally, sample names should be kept consistent across all online databases and physical resources (e.g., museum collections or project-specific sample archives).
Animal ID	String		A researcher-generated unique ID for the individual animal from which the sample was collected: usually a unique string of both characters and integers (e.g., “BZ19-114” to indicate animal 114 sampled in 2019 in Belize). Ideally, animal names should again be kept consistent across online databases and physical resources. Can be left blank in cases where animals are not individually identified (e.g., pooled mosquito testing).
Latitude	Number	**✓**	Latitude of the collection site in decimal format. Equivalent to dwc:decimalLatitude.
Longitude	Number	**✓**	Longitude of the collection site in decimal format. Equivalent to dwc:decimalLongitude.
Spatial uncertainty	Number		Coordinate uncertainty from GPS recordings, post-hoc digitization, or systematic alterations (e.g., jittering or rounding) expressed in meters. Equivalent to dwc:coordinateUncertaintyInMeters.
Collection day	Integer		The day of the month on which the specimen was collected. Equivalent to dwc:day.
Collection month	Integer		The numeric month in which the specimen was collected. Equivalent to dwc:month.
Collection year	Integer		The year in which the specimen was collected. Equivalent to dwc:year.
Sample collection method	String	**✓**	The technique used to acquire the sample and/or the tissue from which the sample was acquired (e.g. “visual inspection”; “swab”; “wing punch”; “necropsy”).
Sample collection body part	String		Part of the animal body that samples are generated or collected from (e.g., “rectum”; “wing”).
Sample material	String		Organic tissue or fluid being collected (e.g., “liver”; “blood”; “skin”; “whole organism”).

Equivalent Darwin Core terms are noted in the descriptor. Data types align to those used in the JSON Schema specification.

**Table 2:** Data standard field definitions (part 2): host identification and traits.

Variable	Type	Required	Descriptor
Host identification	String	**✓**	The Linnaean classification of the animal from which the sample was collected, reported at the lowest possible level (ideally, species binomial name: e.g., “Odocoileus virginianus” or “Ixodes scapularis”). As necessary, researchers may also include an additional field indicating when uncertainty exists in the identification of the host organism (see “Adding new fields”). Equivalent to dwc:scientificName.
Organism sex	String		The sex of the individual animal from which the sample was collected. Equivalent to dwc:sex.
Live capture	Boolean		Whether the individual animal from which the sample was collected was alive at the time of capture. Should be TRUE or FALSE; lethal sampling should be recorded as TRUE as this field describes the organism at the time of capture.
Host life stage	String		The life stage of the animal from which the sample was collected (as appropriate for the organism) (e.g., “juvenile”, “adult”). Equivalent to dwc:lifeStage.
Age	Number		The numeric age of the animal from which the sample was collected, at the time of sample collection, if known (e.g., in monitored populations).
Age units	String		The units in which age is measured (usually years). Should always be provided if age is provided.
Mass	Number		The mass of the animal from which the sample was collected, at the time of sample collection.
Mass units	String		The units that mass is recorded in (e.g., “kg”). Should always be provided if mass is provided.
Length	Number		The numeric length of the animal from which the sample was collected, at the time of sample collection.
Length measurement	String		The axis of measurement for the organism being measured (e.g., “snout-vent length”; “wing length”; “primary feather”). Should always be provided if length is provided.
Length units	String		The units that length is recorded in (e.g., “meters”). Should always be provided if length is provided.
Organism quantity	Number		A number or enumeration value for the quantity of organisms. Equivalent to dwc:organismQuantity.
Organism quantity units	String		The units that organism quantity is recorded in (e.g. “individuals”, “kg”). Should always be provided if organism quantity is provided. Equivalent to dwc:organismQuantityType.

Equivalent Darwin Core terms are noted in the descriptor. Data types align to those used in the JSON Schema specification.

**Table 3:** Data standard field definitions (part 3): detection methods and parasite identification.

Variable	Type	Required	Descriptor
Detection target	String	**✓**	The taxonomic identity of the parasite being screened for in the sample. This will often be coarser than the identity of a specific parasite identified in the sample: for example, in a study screening for novel bat coronaviruses, the entire family *Coronaviridae* might be the target; in a parasite dissection, the targets might be Acanthocephala, Cestoda, Nematoda, and Trematoda. For deep sequencing approaches (e.g., metagenomic and metatranscriptomic viral discovery), researchers should report each alignment target used as a new “test” to maximize reporting of negative data, or alternatively, select a subset that reflect specific study objectives and the focus of analysis (e.g., specific viral families). Equivalent to dwc:associatedOccurrences.
Detection method	String	**✓**	The type of test performed to detect the parasite or parasite-specific antibody (e.g., “PCR”, “ELISA”).
Forward primer sequence	String		The sequence of the forward primer used for parasite detection (e.g., for a pan-coronavirus primer: 5’ CDCAYGARTTYTGYTCNCARC 3’). (Strongly encouraged if applicable, e.g., for PCR.)
Reverse primer sequence	String		The sequence of the reverse primer used for parasite detection (e.g., 5’ RHGGRTANGCRTCWATDGC 3’). (Strongly encouraged if applicable, e.g., for PCR.)
Gene target	String		The parasite gene targeted by the primer (e.g., “RdRp”, e.g., for PCR.).
Primer citation	String		Citation(s) for the primer(s) (ideally doi, or other permanent identifier for a work, e.g. PMID).
Probe target	String		Antibody or antigen targeted for detection. (Strongly encouraged if applicable, e.g., for ELISA.)
Probe type	String		Antibody or antigen used for detection. (Strongly encouraged if applicable, e.g., for ELISA.)
Probe citation	String		Citation(s) for the probe(s) (ideally doi, or other permanent identifier for a work, e.g. PMID).
Detection outcome	String	**✓**	The test result (i.e., “positive”, “negative”, or “inconclusive”). To avoid ambiguity, these specific values are suggested over numeric values (“0” or “1”). Equivalent to dwc:occurrenceStatus.
Detection measurement	Number		Any numeric measurement of parasite detection that is more detailed than simple positive or negative results (e.g., viral titer, parasite counts, sequence reads).
Detection measurement units	String		Units for quantitative measurements of parasite intensity or test results (e.g., “Ct”, “TCID50/mL”, or “parasite count”).
Parasite identification	String	**✓**	The identity of a parasite detected by the test, if any, reported to the lowest possible taxonomic level, either as a Linnaean binomial classification or within the convention of a relevant taxonomic authority (e.g., “Borrelia burgdorferi” or “Zika virus”). Parasite identification may be more specific than detection target.
Parasite ID	String		A researcher-generated unique ID for an individual parasite (primarily useful in nested cases where this ID is used as an animal ID in another row, such as pathogen testing of a blood-feeding arthropod removed from a vertebrate host).
Parasite life stage	String		The life stage of the parasite from which the sample was collected (as appropriate for the organism) (e.g., “juvenile”, “adult”).
GenBank accession	String		The GenBank accession for any parasite genetic sequence(s). Accession numbers or other identifiers for related data stored on another platform should be added in a different field (e.g. GISAID Accession, ImmPort Accession). Equivalent to dwc:otherCatalogNumbers.

Equivalent Darwin Core terms are noted in the descriptor. Data types align to those used in the JSON Schema specification.

Many of the fields are open text, and this flexibility is intentional. The diversity of collection, detection, and measurement methods that researchers use is likely to be beyond the scope of a single controlled vocabulary. Restrictive values may therefore limit the adoption of the data standard by the community. To that end, we have elected to leave these fields as open text in this version of the data standard, but may restrict values as the standard matures. Nevertheless, we encourage users to take advantage of existing controlled vocabularies (see Supporting Information) when using this standard.

In Table 4, we show how a real, previously published dataset^40^ could be formatted using the data standard. The example dataset describes a single vampire bat (BZ19-114) tested for coronaviruses in Belize in 2019: a rectal swab tested negative, while an oral swab tested positive, leading to the identification of a novel alphacoronavirus. All mandatory and relevant fields are shown, and cells are left blank if they do not apply (e.g., parasite identity is always empty for negative test results). The data in Table 4 are only a subset of the full dataset, which is shared in full on the PHAROS platform (project: prjRPayEvMecN). While project-level metadata will likely be captured upon deposit in a scientific data repository, we include metadata for the example project in Table S4 (see Supporting Information).

**Table 4:** An example dataset describing test results for two samples collected from one animal, documented using the minimum data standard. This table is divided into three parts that correspond to data standard field definitions (Tables 1–3). In practice, this would be a single table with two rows (see Supplemental File 3).

Data table part 1 (see definitions in Table 1)									
	Sample ID	Animal ID	Latitude	Longitude	Collection day	Collection month	Collection year	Sample collection method	Sample collection body part
1	OS BZ19-95	BZ19-114	17.7643	−88.6521	23	04	2019	Swab	Mouth
2	RS BZ19-95	BZ19-114	17.7643	−88.6521	23	04	2019	Swab	Rectum

Data table part 2 (see definitions in Table 2)						
	Host identification	Organism sex	Live capture	Host life stage	Mass	Mass units
1	Desmodus rotundus	male	TRUE	subadult	0.023	kg
2	Desmodus rotundus	male	TRUE	subadult	0.023	kg

Data table part 3 (see definitions in Table 3)							
	Detection target	Detection method	Gene target	Primer citation	Detection outcome	Parasite identification	GenBank accession
1	Coronaviridae	semi-nested PCR	RdRp	10.3390/v9120364	positive	Alphacoronavirus	OM240578
2	Coronaviridae	semi-nested PCR	RdRp	10.3390/v9120364	negative		

### How to use the data standard

For researchers who want to apply the data standard to their own projects, we recommend following four basic steps:

1. **Fit for purpose**. The dataset or data to be collected describe wild animal samples that were examined for parasites. Each record must include the host identification, diagnostic methods used to identify parasites, outcome of the diagnostic method, parasite identification, and the date and location of sampling.
2. **Tailor the standard**. Researchers should consult the list of fields in Tables 1–3 and identify (i) which fields beyond the required fields are applicable to their study design, (ii) which ontologies or controlled vocabularies may be appropriate for free text fields, and (iii) whether additional fields are needed.
3. **Format the data**. Template files in.csv and.xlsx format are available in both the supplement of this paper and from GitHub (github.com/viralemergence/wdds).
4. **Validate the data**. We have provided both a JSON Schema that implements the standard, and a simple R package (available from GitHub at github.com/viralemergence/wddsWizard) with convenience functions to validate data and metadata against the JSON Schema.
5. **Share the data**. Researchers should make their data available in a findable, open-access generalist repository (e.g., Zenodo) and/or specialist platform (e.g., the PHAROS platform).

We discuss best practices for some of these steps in greater depth below.

### Best practices for flexibility and extensibility

**Fig. 1:**
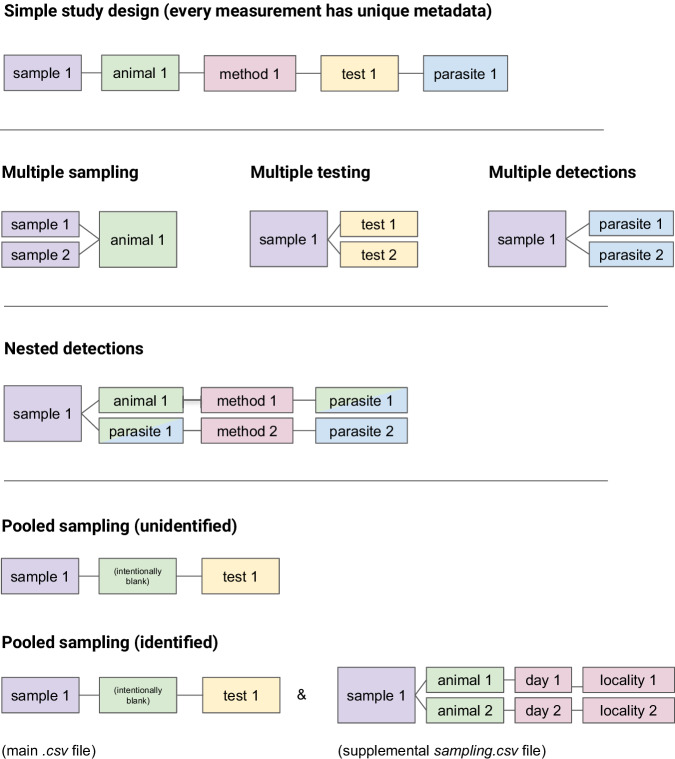
Examples of one-to-one, many-to-one, and one-to-many relationships between fields of the minimum data standard, including commonly-encountered “special cases.” In a simple study design (top row), one sample corresponds to one animal, one sampling method, one parasite test, and potentially, one parasite detection. However, in other studies, multiple samples may be collected from the same animal (e.g., blood and wing punch collected from a bat), a single sample may be tested multiple times (e.g., the blood sample is screened for both coronaviruses and paramyxoviruses), or multiple parasites may be detected in one sample (e.g., the blood sample tests positive for a coronavirus and a paramyxovirus) (second row). Nested detections (third row) can occur when a parasite associated with one animal itself harbors another parasite (e.g., a flea is sampled from a rat, and the flea also tests positive for *Yersinia pestis*). Researchers may also combine samples from multiple animals into a single pooled sample (bottom row). In some cases, the associated animals are “unidentified” (e.g., a pooled sample of 30 mosquitoes). However, if a researcher does have data on each animal linked to a pooled sample, they can provide it in an additional file.

Although our data standard is intended to capture a minimal set of information, not all fields are applicable to every study design. For example, studies that use PCR as a diagnostic method have different applicable fields (“Forward primer sequence,” “Reverse primer sequence,” “Gene target,” “Primer citation”) than those using ELISA (“Probe target,” “Probe type,” “Probe citation”; see Table 3). Similarly, some studies that use a pooled testing approach may leave the “Animal ID” field blank, because animals are not individually identified by researchers (e.g., testing of mosquito pools for arboviral diseases); in other cases, a pooled test may be linked to multiple Animal ID values, and researchers can provide associated metadata on individual animals in a supplemental file (see Fig. 1).

Some datasets may not be able to meet a comprehensive standard for documentation. When data are missing or fields are inapplicable, researchers should leave fields or cells blank instead of using placeholder values like “NA”^41^. For example, in some projects, limited funding or study protocols may preclude all captured animals from being sampled or all samples from being tested. Researchers might therefore include a mix of records of animals or samples with no attached test data (i.e., leaving “Detection outcome” blank). Similarly, archival samples that are rescued from old projects, or older museum specimens that are sampled for parasites^42^, may not always have complete date information, leading to “Collection day” and “Collection month” being left blank. We encourage researchers to adapt our data standard to their specific purposes and, as appropriate, to consider sharing their data in multiple applicable formats. For example, in the previous example, researchers might choose to both share their test results on the PHAROS platform and share a more comprehensive record of all sampling on Zenodo.

Researchers may also wish to include additional fields beyond the minimum data standard to share other kinds of information. For example, researchers might add fields for “Health status” (example values: “healthy”; “sick”; “injured”) or “Reproductive status (“pregnant”; “lactating”), or might use an an all-purpose “Notes” column to flag unusual records or non-standardized information about sampling (e.g., the circumstances under which a dead animal was found, such as opportunistic roadkill collection). Similarly, in cases where findings are particularly sensitive for public health or economic reasons, researchers might consider including some guidance on how to interpret them in the data itself. For example, the data shared by the USAID PREDICT 2 project includes a field called “Interpretation,” which provides guidance such as this disclaimer on a positive test result: “[The virus detected in this sample] is the known ebolavirus, Bombali virus, detected in an Angolan free-tailed bat. This virus has previously been found in bats in Sierra Leone as part of the PREDICT project. Further characterization is ongoing to understand the zoonotic potential of this virus.”

### Best practices for sharing (and withholding) data

When using the data standard, we suggest that researchers should follow scientific conventions and best practices for data science, such as: reporting measurements in metric units; reporting taxonomic information at the most granular level possible for both the host and parasite; and leaving empty and non-applicable cells blank, rather than assigning a placeholder such as “NA”^41^. Researchers should also ensure that their manuscript comprehensively describes all important aspects of sampling methodology, such as the circumstances (e.g., systematic and planned sampling versus opportunistic collection of unusual carcasses), how animal taxonomy was determined (e.g., expert opinion based on morphology versus DNA barcoding), and how samples were prepared (e.g., specific products or kits used, or specific details about the methods used in parasitological dissections). These details will often be the same for each individual row of data, so we exclude them from the template. However, interpreting a study’s data correctly may still depend on these data being available. Researchers should also ensure that their study documents any relevant epidemiological observations (e.g., unusual disease presentation or nearby indicators of human-wildlife contact such as hunting traps, farms, or sewage discharge). Finally, whenever possible, researchers should also share all sequence data in an open repository.

As with other kinds of biodiversity data^43,44^, sharing wildlife disease data paired with high-resolution location data can sometimes be unsafe or inadvisable. For example, sharing the location of a bat roost where viruses have been detected may lead to animal culling, which in turn increases the risk of viral exposure for local human communities^45,46^. There may also be biosafety or biosecurity risks associated with location data, depending on the characteristics of the parasite in question; for example, anthrax spores can persist at a carcass site for several years^47,48^. In sensitive cases, researchers could consider truncating longitude and latitude values, or, potentially, jittering records with random noise. They should then carefully and clearly document the obfuscation process; guidance on this practice exists for other kinds of biodiversity data^49^. In some cases, this obfuscation may still be insufficient to prevent malicious use^50^. In high-risk cases, journal editors should work closely with authors to ensure that neither the manuscript itself nor any supplementary data have a significant potential to cause harm.

### Best practices for publishing datasets

Published data should be stored in commonly used, non-proprietary flat file formats, like comma-separated values (i.e.,.csv with UTF-8 encoding and a period decimal separator), to increase accessibility, interoperability, and utility. Non-proprietary file formats increase access by removing the requirement to have a particular piece of software to open a file. Formats like .csv can also be used across all major operating systems, programming languages, and scientific analysis software suites, greatly expanding interoperability and utility.

The data deposit should contain sufficient documentation to facilitate discovery and use by researchers outside of the project. Data contributors can take steps to increase data discoverability by providing complete project metadata. Using persistent identifiers (PIDs) to create explicit links between the dataset and related publications via digital object identifiers (DOI), individuals with Open Researcher and Contributor IDs (ORCID), organizations with Research Organization Registry (ROR) identifiers for institutional affiliations, and funders with CrossRef Funder identifiers for funding sources creates strong semantic links that improve search results and allow for automated indexing of relationships. Our approach to project-level metadata is based on the DataCite Metadata Schema^29^, and includes fields recommended by the Generalist Repository Ecosystem Initiative^30^ to maximize data discoverability and metadata interoperability. Much of this metadata, if not more, will be captured upon deposit in scientific repositories.

Researchers must be able to interpret the data in order to use it appropriately. To that end, it is important that data contributors include a written description of the data, its intended use, and known limitations (e.g., explanations of missing values or fields) in the project metadata, as well as a data dictionary describing the fields of the flat data file. By using a data standard, data producers can quickly create a data dictionary. To ensure this data standard remains interoperable with other data initiatives, we provide cross-mapping of the fields to the Darwin Core terms^51^ used for biodiversity observations, as well as links to different GenBank data products through unique identifiers. These fields are validated automatically when using the Wildlife Disease Data Standard JSON Schema through the wddsWizard R package. For further specificity, data producers may use terms from ontologies or controlled vocabularies when referring to specific measurements or tests

To ensure that data producers get credit for their work, data should be deposited into archival platforms that can provide a PID like a DOI, capture project metadata, and surface relevant works via search. Commonly used archives include Zenodo, OSF.io, DataDryad, and figshare. Some journals have agreements with archival data platforms that can waive the costs of archiving data, in addition to creating a semantic link between the DOI of the publication and the DOI of the dataset.

Data producers are encouraged to deposit material in multiple archives, including discipline-specific and generalist repositories. Publishing the flat files on multiple data platforms has a series of advantages. First, increasing the number of copies decreases dependency on a single platform, increases data longevity, and reduces the risk of deletion or modification. Second, having data on multiple platforms (and especially discipline-specific platforms) maximizes the chances that they are discovered. Finally, for data contributors, depositing data in general-purpose repositories also offers additional flexibility in terms of archiving record- or project-level information that is not in the scope of our data standard. For example, the ImmPORT platform uses a data model that allows researchers to provide direct links to NIH resources, detailed lists of personnel involved in a project, and direct connections to relevant biomedical ontologies^52^.

## Discussion

Here, we propose a data standard for wildlife infectious disease studies. With minimal modifications, the same template could also be used for related types of data, such as records of plant pathogens, or infections in captive animal populations such as zoos and wildlife sanctuaries. However, other types of spatiotemporal disease data may already have associated best practices and dedicated or otherwise well-suited repositories. For example, disaggregated but carefully de-identified human infectious disease data can be shared in epidemic settings on the Global.health platform^53^; host, vector, and parasite occurrence data can also all be documented in Darwin Core format and shared in GBIF^54–56^.

We encourage researchers to adopt this minimum standard, and to deposit their data in generalist repositories (e.g., Figshare, Data Dryad, or Zenodo) and specialist platforms (e.g., PHAROS), so that their data are findable, accessible, interoperable, and reusable (FAIR) by other scientists^16^. Doing so will help researchers meet the minimum requirements for data sharing now adopted by most journals and scientific funders. Researchers could even consider sharing data before or independent of manuscript publication, especially in cases where negative data might not be publishable, or where timely sharing of findings might be particularly relevant to public health or conservation. Progress toward open, timely data sharing will make wildlife disease research a richer and more rigorous field, leading to better insights about emerging threats to human and animal health.

## Supplementary information

Supplemental File 1

Supplemental File 2

Supplemental File 3

Supplemental File 4

## Data Availability

The example dataset and blank templates are available from GitHub at github.com/viralemergence/wdds.

## Code availability

An R package to validate data against the data standard described in this paper is available from GitHub at github.com/viralemergence/wddsWizard.
